# Image Augmentation Techniques for Mammogram Analysis

**DOI:** 10.3390/jimaging8050141

**Published:** 2022-05-20

**Authors:** Parita Oza, Paawan Sharma, Samir Patel, Festus Adedoyin, Alessandro Bruno

**Affiliations:** 1 Computer Science and Engineering Department, School of Technology, Pandit Deendayal Energy University, Gandhinagar 382007, India; paawan.sharma@sot.pdpu.ac.in (P.S.); samir.patel@sot.pdpu.ac.in (S.P.); 2 Department of Computing and Informatics, Bournemouth University, Poole BH12 5BB, UK; fadedoyin@bournemouth.ac.uk

**Keywords:** data augmentation, deep learning, medical imaging, mammograms

## Abstract

Research in the medical imaging field using deep learning approaches has become progressively contingent. Scientific findings reveal that supervised deep learning methods’ performance heavily depends on training set size, which expert radiologists must manually annotate. The latter is quite a tiring and time-consuming task. Therefore, most of the freely accessible biomedical image datasets are small-sized. Furthermore, it is challenging to have big-sized medical image datasets due to privacy and legal issues. Consequently, not a small number of supervised deep learning models are prone to overfitting and cannot produce generalized output. One of the most popular methods to mitigate the issue above goes under the name of data augmentation. This technique helps increase training set size by utilizing various transformations and has been publicized to improve the model performance when tested on new data. This article surveyed different data augmentation techniques employed on mammogram images. The article aims to provide insights into basic and deep learning-based augmentation techniques.

## 1. Introduction

**Figure 1:**
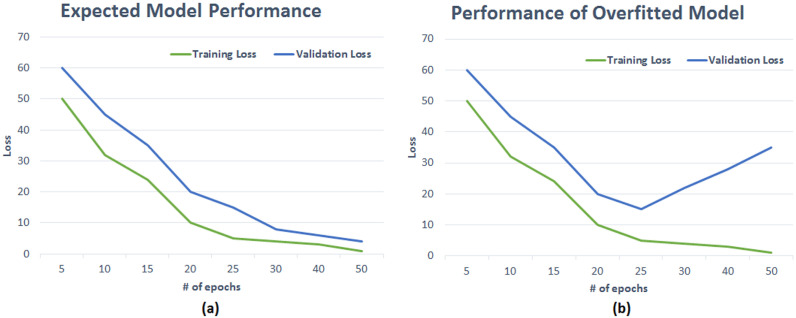
(**a**) Shows the ideal trend of the model with training and validation error functions decreasing almost simultaneously. (**b**) Shows the undesired effect of overfitting, having the training error decrease and, conversely, validation error increases suddenly.

**Figure 2:**
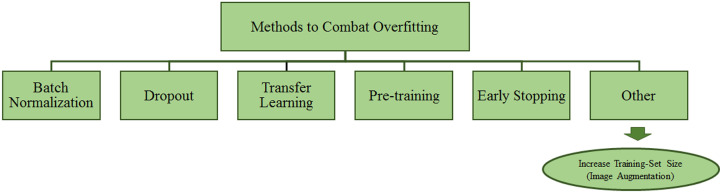
Methods to tackle overfitting.

Amongst various artificial intelligence fields, Deep Learning (DL) is widely adopted for the processing and analysis of radiological images. DL has been successfully applied to multiple Computer Vision tasks such as Object Segmentation, Detection, and Classification, mainly thanks to accuracy rates achieved by convolutional neural networks (CNNs). CNNs have the capabilities to automatically learn features through several network layers from a large set of labelled datasets [1]. Concerning the biomedical image analysis topic, CNNs have been successfully utilised for various tasks such as lesion or tumour classification, suspicious region detection, and abnormality detection [2,3,4]. DL-based solutions serve as a second opinion tool for expert radiologists and assist them in decision-making, and proper treatment planning [5]. However, there needs to be a large amount of ground truth to build a DL model capable of inferring knowledge from data and avoiding the model being very accurate only on the training dataset images. The latter goes under the name of overfitting [6,7] and represents a critical issue to overcome to have a model capable of delivering appropriate knowledge inference capabilities on a given application domain. Furthermore, having high-quality and manually annotated data is a time-consuming and expert dependent task. Unfortunately, that is quite common in the context of mammogram analysis [8,9,10]. One of the most challenging tasks for DL models is the generalisation, with generalisation being the capability of models to recognise those categories they were trained for on new data [11,12]. The model with poor generalisation generally does not perform well due to high overfitting on the training set. Overfitting can be observed somehow in the plot showing validation accuracy at every epoch of the training phase [1]. Figure 1 shows the pictorial representation of models with and without overfitting. The training and validation loss curve is progressively and simultaneously reducing, which is a perfect circumstance, as shown in Figure 1 (left). The right side of the figure shows overfitting, in which the validation loss begins to grow after a certain number of epochs. In contrast, the training loss keeps decreasing. That is due to the model’s inability to work effectively with unknown or new data. One of the reasons for this phenomenon might be a lack of enough training samples. The validation error of suitable DL models should continue to decrease along with the training error. Data augmentation methods can help achieve this task. Augmented data can characterise the inclusive set of input data points and minimise the distance between validation and training data. Data augmentation techniques apply alterations to training datasets to produce more samples. Moreover, this technique helps the model avoid learning features too specific to the original data, resulting in a more generalised model with improved performance on the test dataset. Class distribution imbalance in datasets is another common challenge. For instance, binary classification problems occur when one class (the minority class) holds considerably fewer samples than the other class (the majority class). Due to this, the model may get biased towards the majority class, possibly resulting in misclassification. Augmenting the minority class images may be used to mitigate the imbalance problem. Data augmentation is not the only approach to reduce the effect of overfitting and class imbalance. Other options for avoiding overfitting in DL models are also explored in the literature (see Figure 2).

Batch Normalization: Batch Normalization can overcome the side-effect of overfitting by diminishing the internal covariate shift and instability in the distributions of Deeper networks’ layer activations. For each mini-batch, batch normalisation standardises the inputs to a layer. That has the effect of bringing the learning process into balance.

Dropout: Dropout applies during the training phase to get randomly selected neurons ignored. That avoids the so-called layer’s “over-reliance” on a few inputs. Still, apart from that, it also prevents neurons from co-adapting to training data.

Transfer Learning (TL): Transfer learning improves models’ performances on new and unknown data. The main point with TL is employing pre-trained models to be fine-tuned on a specific application domain using a small-sized dataset.

Pre-training: Model pre-training is similar to TL; the only difference is that model architectures can be defined, and weights are transferred.

Early-stopping: It allows providing an arbitrarily large number of training epochs to suddenly stop training if the model does not perform well on the validation set.

### 1.1. Research Contribution

**Figure 3:**
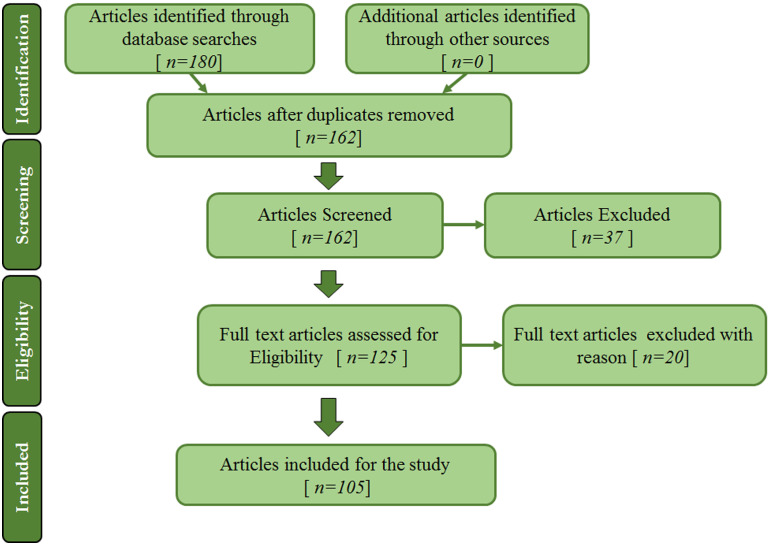
The PRISMA flow diagram and the selection method.

Image augmentation techniques have been applied to mammogram datasets to increase the training set size, allowing data-hungry learners to benefit from more representative data. A review is conducted to summarise image augmentation techniques used in medical imaging applications such as deep learning-based for breast cancer diagnosis. The two following main categories of image augmentation techniques are surveyed here: (1) Basic image augmentation techniques and (2) Advanced augmentation techniques. The search terms used in the study are combinations of keywords such as “data augmentation”, “image augmentation”, “deep learning”, “breast cancer”, and “mammograms”. Articles that do not utilise or discuss data or image augmentation were discarded in this study. The research mainly focuses on image augmentation for mammogram images. Therefore, articles whose subject is on other imaging modalities such as CT scans, Breast MRI, Breast ultrasounds, Histopathology, etc., are excluded. Articles on image augmentation used in the literature for breast image analysis applications are also summarised according to the dataset, model, technique, tasks performed, etc. In the scientific literature, comprehensive and insightful surveys on image augmentation methods are present; some are specific to medical images. For example, the authors in [1] suggested several data augmentation solutions as ways to tackle models overfitting due to low-sized datasets. Another article [5] presents a thorough evaluation of the data augmentation methods employed in the broad topic of medical image analysis. In further detail, the authors focused on CT and MRI. However, another article reports recent advancements in data-augmentation techniques for brain MRI [13] by examining the papers submitted to the Multimodal Brain Tumor Segmentation Challenge (BraTS 2018 edition [14]). This paper aims to quickly access the research field and form an appropriate groundwork for the domain. This work examines several articles from various conferences, books and indexed journals out of scientific databases such as Scopus, IEEE, Web of Science and PubMed in compliance with PRISMA (Preferred Reporting Items for Systematic Reviews and Meta-Analyses) [15] recommendations. Figure 3 depicts the selection procedure. From a data augmentation standpoint, the goal of this work is to provide insights into the broader area of mammography image analysis. Mammograms are the primary matter of discussion in the article. The goal is to give readers an understanding of basic and deep learning-based augmentation approaches. Therefore, other than other review articles on the topic, the main goal here is to survey different approaches for data augmentation and check through the pros and cons of mammogram analysis related tasks. The impact of data augmentation techniques is analysed by checking through the dataset size increase and the pre and post-augmentation accuracy rates of models over a specific task.

### 1.2. Paper Topology

The paper is structured as follows: Section 1 provides background and context for image augmentation within the broad topic of a deep learning-based CAD system for medical imaging. Section 2 delves into various image augmentation techniques used in practice. Advanced image augmentation methods are showcased in Section 3. Insights into test-time augmentation are provided in Section 4. Discussions and conclusions, Section 5 and Section 6, respectively end the paper.

## 2. Basic Image Augmentation Techniques

Data augmentation techniques have been used to increase the size of the training set to provide more illustrative training samples to large-capacity learners [16]. Data augmentation encompasses a comprehensive range of techniques by inserting random variations into the existing training samples while preserving class labels. The purpose of data augmentation is to improve the model knowledge inference capability. One of the most meaningful principles adopted in data augmentation relates to the physical phenomenon of a state perturbation. The latter takes the form of slightly changed versions of images. Consequently, it increases the dataset size, allowing the network to infer knowledge from a broader pool of images. Therefore, when using deep learning in computer vision tasks, three types of data augmentation are the most likely; (1) Dataset generation and expansion. (2) On-the-fly data augmentation. (3) Amalgamation of Dataset generation and on-the-fly data augmentation. As already mentioned and widely covered in the scientific literature, supervised DL models [17] need a large amount of training data to unleash their knowledge inference capabilities fully. In the worst-case scenario, only one image is available, and data augmentation comes into play to produce a complete image collection. The task carries out random transformations (rotation, flipping, etc.) and other effects on the original image. Then, the newly generated images feed the DL model during the training phase. Methods like generation and expansion can forge N number of images. However, these approaches are not exempt from flaws: employing images generated by these methods does not necessarily improve models’ generalisation abilities. On-the-fly data augmentation (sometimes also called in-place) is the second type of data augmentation [18]. On-the-fly data augmentation helps DL model training see new variations of images at each epoch. It takes image batches as input and then applies a series of random transformations and other effects on each image in the batch. It finally returns a randomly altered image batch.

### 2.1. Geometric Transformations

In geometric transformation, an original image undergoes various modifications such as translation, rotation, scaling, flipping, or resizing to increase the training dataset size [5]. These conventional data augmentation techniques produce somewhat correlated images [19] and hence offer significantly less improvement to the model training and generalisation over test data. However, these transformations lead to a significant increase in the training dataset; therefore, they are widely used in the domain [13]. This section presents the most commonly used geometric transformations for computer-aided breast cancer diagnosis. It also briefly surveys methods building on the data augmentation methods mentioned above.

#### 2.1.1. Flipping

Flipping generates a mirror image of an image with both horizontal or vertical axes. The horizontal axis is more preferred over vertical flipping because the top and bottom parts of an image may not be interchangeable always [13]. However, flipping cannot always be a label-preserving transformation (e.g., MNIST dataset) [1]. For example, in datasets such as DDSM [20] and CBIS-DDSM [21], most of the breast profiles are on the left side of the mammograms. Making uniform direction of the breast in mammograms makes padding easier to perform during preprocessing steps.

#### 2.1.2. Rotation

Images are rotated leftward or rightward across an axis within the range [1°, 359°]. The rotation angle determines the safety of this augmentation technique. The possibility of keeping the label post-transformation is known as a Data Augmentation method’s safety. An image label might no longer be preserved with an increase in rotation degree. For example, rotation transformation is possibly safe on medical image datasets (X-ray, mammograms, Breast MRI, etc.) as well as on images of other datasets like ImageNet [22], but not on images of 9 and 6 for digit identification tasks.

#### 2.1.3. Translation

Translation applies to prevent positional bias [1]. This transformation translates the whole image by a given translation vector along a specific direction. It helps the network learn geographically invariant properties rather than focusing on features present in a single spatial location [13]. In the case of breast mammograms, translation of images can generate suitable augmented images. After the translation, padding or pixel replication usually comes into play to fill out the leftover space. The process keeps the image dimensions [1].

#### 2.1.4. Scaling

**Figure 4:**
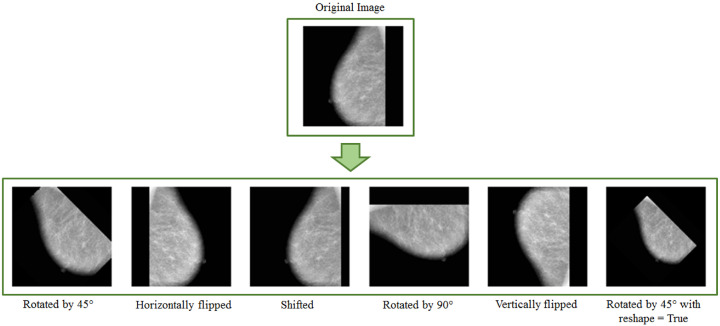
Example of images after applying geometric transformation.

Scaled versions of images are added to the training set; deep neural networks can learn features regardless of their original scale. Furthermore, scaling can be applied using scaling factors in different directions. For example, breast lesions may vary in size; this transformation can bring realistic augmented images into the training dataset. Figure 4 shows examples of geometric transformations applied to MIAS [23] images.

Limited dataset size is one of the most common barriers in the medical research domain. Therefore, the scientific literature provides a wide range of techniques to handle this issue. For example, Costa et al. [24] employed data augmentation to create new images based on their original clinical mammography dataset, and they compared the results across different CNN architectures. The authors used geometric transformations such as rotation by varying degrees, flipping and adding Poisson noise. The model performs better when more regions of interest are added to the training step using data augmentation techniques. A new CNN model for identifying architectural distortion is proposed by Oyelade et al. in [8], which uses rotation, flipping, shearing and scaling for data augmentation. In their article, Cha et al. [25] stated, “Deep learning algorithms can improve performance by expanding their training set with synthetic examples”.

Horizontal and vertical flipping methods were used by Omonigho et al. [26] to augment the training set. By augmenting the training set with scaling, horizontal flip, and rotation, the authors could achieve 95.70% overall accuracy on the modified AlexNet model. In another study [27], Rahman et al. showed how specific pre-processing, transfer learning, and data augmentation approaches may help overcome the dataset size bottleneck in medical imaging applications. Geometric transformations such as reflection, translation, random scaling and random rotations were applied to the DDSM mammogram dataset.

Shi et al. [28] implemented a customised CNN to classify BI-RADS [29] density of mammogram images. The MIAS dataset was augmented using various transformations such as zooming, flipping, rotation and shifting. The authors carried out five-fold cross-validation of the model, which yielded an average test accuracy of 83.6%. Still, it is paramount to keep a certain level of variety between the images. Therefore, Khan et al. [30] developed a mammogram classification system and adopted random horizontal and vertical shifts, random shear and zoom as data augmentation techniques.

**Figure 5:**
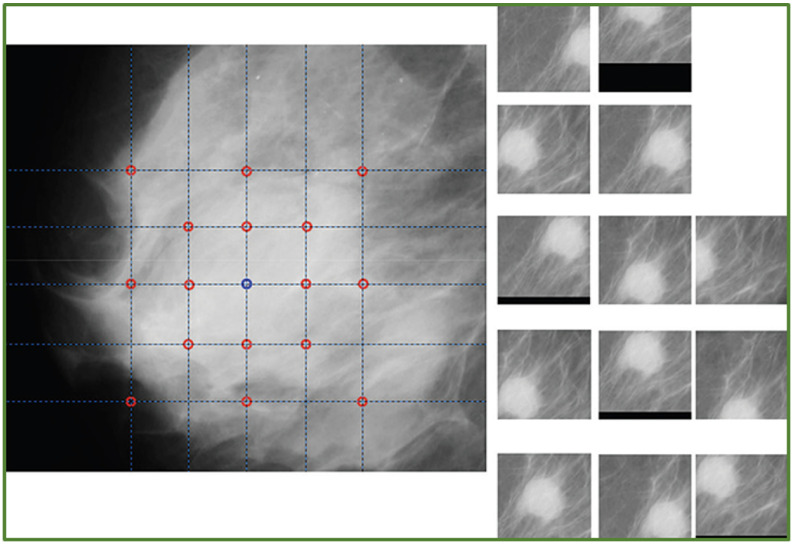
An example of patch of Mammogram and a sample of patches generated with geometric transformation [12].

Zhang et al. [31] performed data augmentation through reflection and rotation. Initially, each original image underwent horizontal flipping, and then original and reflected images were rotated by 90°, 180°, and 270° degrees, respectively. As a result, the dataset increased eight times in size. The authors evaluated seven different architectures and concluded that models built and optimised using data augmentation and transfer learning had a lot of potential for automatic breast cancer detection. Bruno et al. [12] extracted patches from mammogram datasets such as MiniMIAS [32] and their own freely accessible dataset called SuREMaPP. Image transformations such as translations, horizontal reflections, and crops were employed in the study to generate augmented patches. Figure 5 shows an example of patches and augmented patches out of geometric transformations.

Assari et al. [33] suggested a BI-RADS-based CAD system for breast mass discrimination. The study makes use of GoogleNet for transfer learning. The authors used the DDSM breast mammography dataset. The authors used augmentation techniques such as horizontal flipping, clipped ROIs, and random zero-mean Gaussian noise to address the issue of overfitting. Another work by authors of [34] presented a system for mass detection and classification. To address the issue of class imbalance and small-sized datasets, the authors used two different augmentation approaches. In the first method, the authors have augmented the whole dataset and then divided it into training and testing sets.

Muduli et al. [35] ran various geometric transformations and Gaussian noise to generate a large number of samples: 2240 for Inbreast, 3200 for MIAS, and 28,800 for DDSM. The resulting images helped the authors work out the training of a deep CNN for breast cancer classification. A new domain generalization method is proposed in [36] to aid mammography lesion detection techniques. Domain-invariant features were embedded in a range of datasets using a multi-style and multi-view contrastive learning technique. The results showed that the domain generalisation technique is successful and can significantly improve both seen and unseen lesion detection tasks.

### 2.2. Pixel Level Augmentation

Pixel-level augmentation is quite helpful for research in medical imaging fields, as medical images are obtained with several technologies and imaging modalities; hence, they can be essentially assorted in pixel intensities [13]. In pixel-level augmentation, intensities of pixels are perturbed with random noise and a given probability, also called random intensity variation. In addition, a pixel-level augmentation modifies the brightness of an image. Among others, gamma correction (and all its variants), image blurring, and image sharpening represent forms of pixel-level augmentation [37,38,39].

### 2.3. Pseudo-Colour Augmentation

Pseudo-colour augmentation applies to colour channels spaces. Isolating a single colour channel, such as R, G, or B, is the first step for colour augmentation consisting of deriving a colour histogram that describes the image allows further advanced colour augmentations. Mammograms are turned into pseudo-colour pictures to assess the effectiveness of Mask R-CNN. The latter is carried out using multi-scale morphological sifting, which boosts mass-like patterns. Mask R-CNN is then used with transfer learning to detect and segment masses on pseudo-colour images at the same time [40].

### 2.4. Random Erasing

**Figure 6:**
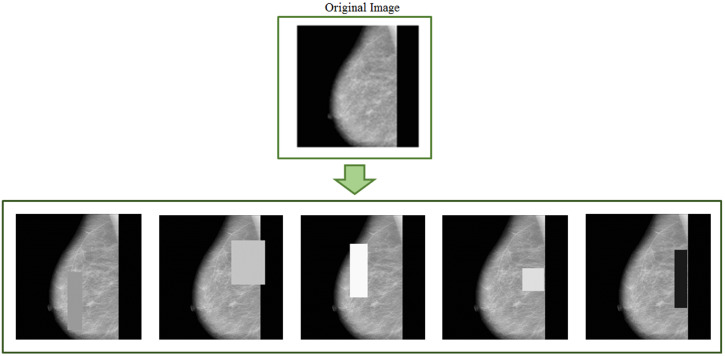
Mammograms after applying random erasing.

Random erasing is another data augmentation technique [41] complementary to the previously described ones. The main goal of this technique is to make a model robust against occlusions in images. One of the most meaningful features is the learning phase being parameter-free. In Figure 6, some examples of random erasing image augmentation are given.

### 2.5. Kernel Filters

Kernel filters rely on spatial filtering techniques to sharpen or smooth pixel values. It uses M×M size filtering masks. Along with transformations such as padding, flipping, and cropping, Kang et al. [42] used a kernel filter swapping pixel values with a n×n sliding window. The experiments were carried out on four different datasets such as SVHN [43], MNIST [44], CIFAR-10 [45], and STL-10 [46]. A different data augmentation method involves combining images by averaging their pixel values. The main point is to generate different samples to have the model be able to generalise information from data [1]. Various kernel filters such as gaussian blur, mean filter, median filter, Laplacian filter, etc., can be employed for data augmentation purposes.

**Figure 7:**
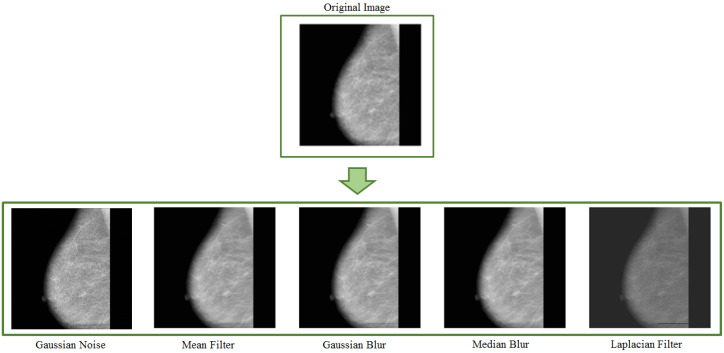
Example of data augmentation based on various filters and noise.

Figure 7 shows some images generated with various kernel filters. Adedigba et al. in [47] employed the augmented dataset to train five state-of-the-art models. The authors used Gaussian blurring and additions of white noise and geometric transformations to increase the training set. The experiments showed DensNet remarkably achieving the highest training and validation accuracy (99.01% and 99.99%, respectively). Artificially generated mammograms and data augmentation techniques are applied by Yemini et al. in [48] to increase and balance the available database at training time. Along with flipping transformation, the authors of this work used Gaussian noise and changed image brightness to generate new images from the original samples.

## 3. Advanced Augmentation Techniques

In the last decade, several methods have been proposed to generate new samples from a reference dataset. The main goal has been to overcome all issues related to basic data augmentation techniques. This section groups advanced techniques for data augmentation into two main categories: GAN-based augmentation, and NST augmentation.

### 3.1. GAN-Based Augmentation

**Figure 8:**
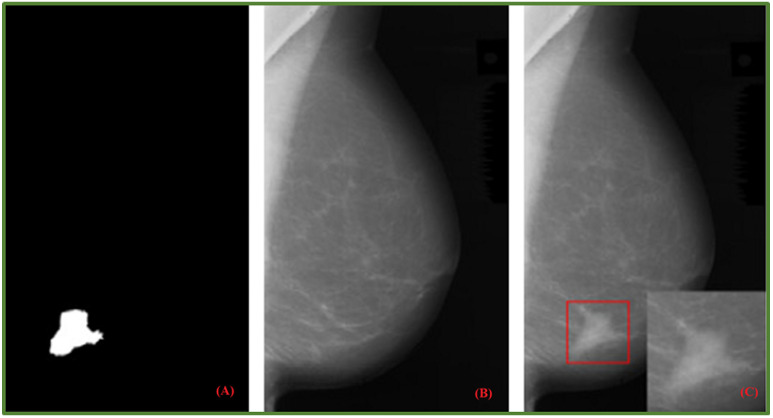
Given mask image (**A**); normal mammogram image (**B**); generated mammogram image with synthetic mask (**C**) [55].

**Figure 9:**
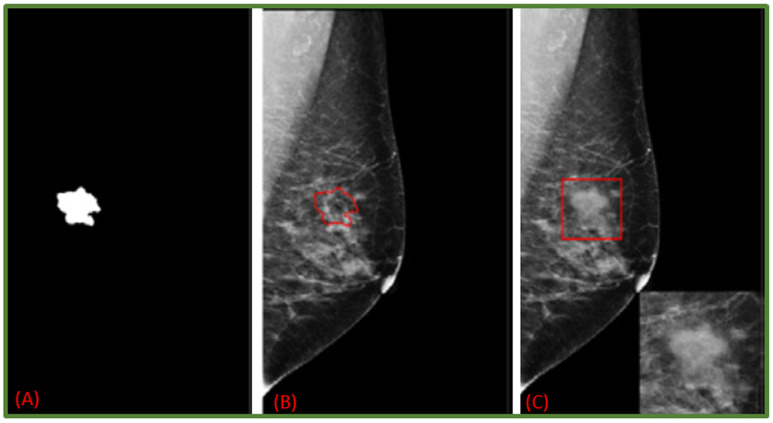
Private Dataset: Given mask image (**A**); normal mammogram image (**B**); generated mammogram image with synthetic mask (**C**) [55].

GANs (Generative Adversarial Networks) belong to the family of unsupervised deep learning algorithms capable of extracting hidden underlying properties from data and employing them in decision-making. The fundamental goal of a GAN is to develop new image samples (by a generator) that the discriminator will not be able to tell apart from the original ones (Both network branches compete against each other and gradually learn to produce better results) [49]. GANs are reliant on two main components, namely, generator and discriminator. Scientific literature shows they are also used to learn noise augmentations. In adversarial training, one model classifies examples while another adds noise to deceive the classifier. The adversarial model is then given a loss function by the classification model, allowing it to improve itself to create better noise. Including images from adversarial training might help models acquire more robust features that are less sensitive to noise distortions. Although it has been proven that using adversarial search to inject noise improves performance in adversarial cases, it is unclear if this is effective for decreasing overfitting. That is still currently an open challenge and has researchers investigating the link between adversarial attack resistance and actual performance on test datasets [1]. In addition, several DL-based augmentation systems employ adversarial training (including GAN-based and other adversarial learning networks) [50,51]. GANs are a widely used data augmentation approach to detect patterns and variances in image samples from the training dataset [52,53]. They have also been used for breast mass detection [53], mass classification [10] as well as mass segmentation [54]. The realistic level of artificially generated images for medical scenarios is still a debate matter [5]. On the other side, Shen et al. [55] provide a unique strategy based on GANs for generating varied mass images to perform contextual infilling by incepting synthetic masses into mammograms. Furthermore, their system automatically annotates the generated mass from patches. As shown in Figure 8, a mammogram image is transformed into a new one containing the incepted mass. Shen et al. carried out similar experiments on a private dataset too (Figure 9). In [56], the authors generated synthetic data via Cycle GAN. They carried out mass classification tests over 412 images: 212 with cancerous mass and 202 with no cancerous mass. The findings revealed that synthetically generated images, along with domain transformation from unrelated masses, could be used to increase the training sample size and improve the mass classification accuracy rates.

**Figure 10:**
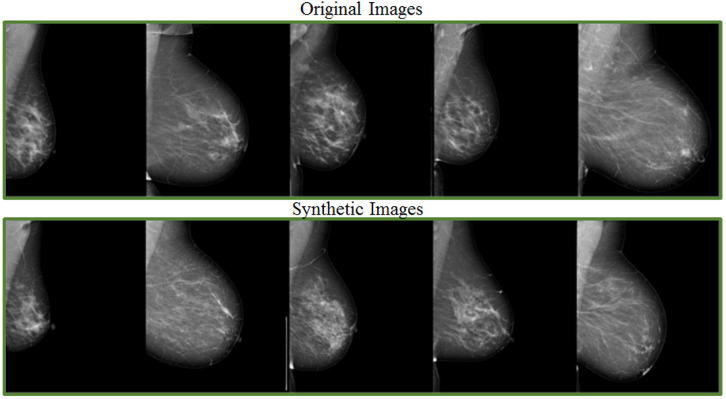
Randomly sampled examples of original and synthetic mammograms [58].

A deep neural system to support tumour recognition in mammograms was proposed in [57], with GANs serving as a data augmentation tool. The research was carried out utilising a large-sized database containing around 10,000 mammographic images from the DDSM dataset. A class-conditional GAN (ciGAN) was trained to perform contextual in-filling, which is subsequently used to synthesise lesions onto healthy screening mammograms in [9]. The authors also showed ciGAN-synthesized samples for cancerous to non-cancerous and non-cancerous to cancerous transformations. Experimental research involving specialist doctors in the assessment of GANs on the generation of medical images was presented in [58]. Some promising results showed that the developments of GAN-based image synthesis could successfully apply to high-resolution medical imaging. Figure 10 provides examples of original and synthetic mammograms from [58]. Users may modify or enrich existing datasets by effortlessly putting a real breast mass or micro-calcification cluster retrieved from a source digital mammography into a different region of another mammogram. Findings of a reader experiment that compared the realism of inserted lesions to clinical lesions were shown in [59]. Using the receiver operating characteristic (ROC) technique, radiologist ratings showed that injected lesions cannot be consistently discriminated from clinical lesions. Swiderski et al. [57] remarked a ResNet-50 classifier trained on GAN-augmented data produced better AUROC than when trained solely with traditionally augmented data. The adoption of data augmentation as an overfitting mitigation technique was investigated in [25]. “In silico” procedural analytic breast and breast mass modelling algorithms were used to create synthetic mammograms. They were then projected into mammographic pictures using simulated X-ray projections.

### 3.2. Neural Style Transfer (NST)

Deep Learning proved effective even in mixing styles out of different images. Neural Style Transfer is meaningfully representative of the quality levels achieved on this [60]. The overall goal is to alter visual representations formed in CNNs [61]. Neural Style Transfer is well known for its uses in creative application domains, but it can also be used to augment data. The technique manipulates the sequential representations across a CNN to transfer the style of one image to another while keeping the original content [1]. Gatys et al. [62] first proposed NST, which typically takes two input images: a content image C to be transferred and a style reference image S. It executes feature learning from the representations of Fl(C) and Fl(S) in layer l of a neural style transfer network [63]. However, suppose the image styles from different datasets are way too far. In that case, it may cause a wide domain gap undermining deep learning models’ capacities to target a specific scenario of interest. Wang et al. [63] proposed a multi-resolution and multi-reference NST network to address style diversity in mammograms. With very high resolution, the network can normalise styles from several vendors (e.g., GE Healthcare (GE) and United Imaging Healthcare (UIH) to the same style baseline.

A novel approach for detecting abnormal and normal regions from mammograms has been presented by Ramadan et al. [64]. They combined a cheat sheet containing standard features retrieved from ROIs with data augmentation boosting CNN performances in breast cancer detection. As a result, the accuracy rate improved by at least 12.2% and precision by at least 2.2%.

Based on the identification of masses in the projections, the authors of [65] assessed the usage of data augmentation and the selection of non-overlapping areas of interest (ROI). Zhang et al. [31] combined data augmentation and transfer learning techniques with CNN models to improve the performance of the classifiers for mammogram images.

### 3.3. Other Techniques

Evolutionary algorithms deal with problem solutions’ optimisations by building on Darwin’s natural selection theory. There are some crossings elements between evolutionary algorithms and the broad pool of machine learning methods for classification, regression, and clustering tasks. In this section, a specific evolutionary algorithm is described for biomedical image augmentation: crossover [66]. The crossover technique is proposed in [66] for medical image classification problems using CNNs. The method creates sample pairs through two-point crossover on already existing training datasets. From N training samples, authors could generate N new samples. The process was examined on the Mini-MIAS dataset with the VGG-16 and VGG-19 pre-trained models. The “natural deformation data augmentation approach” is proposed by Cao et al. [67] as a new data augmentation method based on local elastic deformation. The essential notion is that only the BMass is elastically deformed in a picture containing BMass to replicate the natural changing of BMass, while the local background region in contact with BMass changes accordingly. The research by Chen et al. [68] paved the way for the employability of virtual adversarial training (VAT) to improve the performance of semi-supervised classification of malignant and benign masses from mammograms. This especially applies to medical scenarios with unlabeled medical images. By employing augmentation methods such as Cutout [69] and RandConv [70], Garrucho et al. [71] ran comparisons between eight different object detection models to detect breast masses from mammogram repositories such as OPTIMAM [72], INbreast [73], BCDR [74]. Another study by Tran et al. [75] presented a Transparency strategy-based technique for generating abnormal instances by changing the pixel values. Experiments confirm the proposed approach improving the BI-RADS classification task on mammography assessments. The augmentation method was compared with The CutMix [76] augmentation approach and outperforms the same.

Table 1 summarises basic and advanced augmentation techniques with their strength and limitations. Finally, Table 2 presents a summary of methods that adopted image augmentation strategies to improve the model performance and counter overfitting. Furthermore, we also summarise some articles by highlighting pre and post-augmentation performances as well as pre and post augmentation dataset sizes (see Table 3).

## 4. Test-Time Augmentation (TTA)

**Figure 11:**
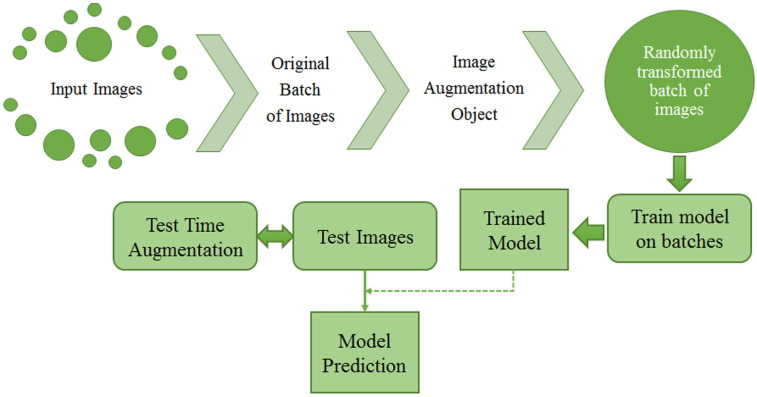
Train and test-time data augmentation.

**Figure 12:**
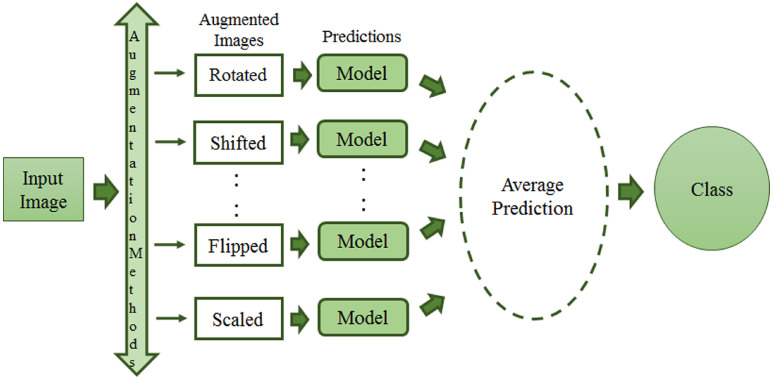
Test-time data augmentation framework.

Over the last few years, a new image augmentation technique has increasingly caught researchers’ interest. It goes under the name of TTA, which stands for Test Time Augmentation. Wang et al. [99] provided the scientific community with a mathematic formulation of TTA. They present TTA as an inference problem with hidden parameters and prior distributions. Therefore, images are considered the results of an elaboration process with hidden parameters. The final goal is to evaluate structure-wise uncertainty associated with image transformations and noise. Other than the previously mentioned techniques, TTA creates various augmented images of the test set, feeds these augmented images to the trained model, and finally returns an ensemble of those predictions to get a more assertive response [100]. Figure 11 shows the process of both train and test time augmentation, while, in Figure 12, test-time data augmentation framework is depicted.

TTA has conveyed new possibilities to the medical imaging field by measuring the strength and network consistency as practical issues [101]. TTA can be used for those methods which modify an incoming example with affine, pixel-level, or elastic transformations in the case of lesion classification from mammograms. The research community has focused on training data augmentations, while data transformation before inference has yet to be fully explored. TTA combines numerous inference findings utilising various data augmentations to categorise one image (see Figure 12). Kim et al. [100] presented a TTA method that is instance-aware and based on a loss predictor. They improved image classification performance with the dynamic use of TTA transformations. The authors of [102] employed TTA for U-Net [103] to tackle medical image segmentation. Another study [104] employed TTA with the model making predictions on five, 224×224 image patches, as well as horizontally reflected patches (for a total of ten patches), and then averaging the outputs on over the ten patches with the softmax layer. An inference approach called Mixup Inference (MI), reliant on simple geometric intuitions was proposed by Pang et al. [105]. The method mixes inputs with additional random samples. Vedalankar et al. [89] addressed the analysis of architectural distortion in mammograms with an integrated solution based on AlexNet and SVM. However, the solution heavily relies on TTA as the data augmentation technique for mammogram images.

## 5. Discussion

This section discusses data augmentation and its employment in mammogram analysis and related tasks. The paper spans the main data augmentation approaches as listed in Table 1. Most of them build on geometric transformations, noise injection, kernel filters, mixing images, random erasing, generative adversarial training, and neural style transfer. In Figure 4, Figure 5 and Figure 7 examples of basic geometric transformations and image filtering show how simple operations and modifications allow increasing datasets’ volumes. Conversely, things gradually become more complex when surveying advanced image augmentation methods. In Figure 8, Figure 9 and Figure 10, pictures respectively taken from Shen et al.’s [55], and Korkinot et al.’s [58] demonstrate the level of sophistication achieved by more recently introduced deep learning techniques such as GANs in generating realistic mammograms. It is not straightforward to discriminate synthetic images from real ones, especially to non-expert eyes. Apart from the considerations mentioned above, it is necessary to span the performances of those models that heavily rely on data augmentation, as shown in Table 2. Around thirty methods tackling tasks such as mammogram classification, suspicious region segmentation and micro-calcification identification are compared across several parameters. The following subsection checks through two main aspects: the data augmentation impact on the dataset sizes, and the pre and post-augmentation performances of the approaches as depicted in Table 2 and Table 3.

### Data Augmentation Impact

Although several methods achieve decent accuracy rates over several datasets, three main points are to be highlighted:

1. Oyelade and Ezugwu [8] achieved a 93.75% accuracy rate on anomaly detection from mammograms using a CNN-based technique and basic data augmentation techniques (rotation by 90, 180 and 270 degrees, mirroring and additive Poisson noise).
2. Conditional infilling GANs for data augmentation in mammogram classification by Dhivya et al. [10] averagely scored 94% accuracy over three different datasets, respectively, MIAS, INBreast and DDSM, which include images having heterogeneous spatial resolution and acquiring device properties. The same method gets to an 88% accuracy rate when only basic data augmentation techniques are adopted.
3. Razali et al. [84] reached an excellent 99% accuracy rate on InBreast and DDSM with basic augmentation techniques on two datasets. However, it would be worth investigating any further improvement with advanced data augmentation techniques. However, after surveying all methods in Table 2, it is noticeable how advanced mammogram augmentation impacts the accuracy rate improvement by 6% over three different datasets. Investigating all elements causing an increase in accuracy on a specific task is not trivial.

Table 3 allows comparing several methods according to the data augmentation impact on dataset size and performances over different datasets. The table consists of three columns for pre-augmentation dataset size, post-augmentation dataset size, and post-augmentation model performance. Overall, regardless of the specific augmentation technique employed, the increasing factor for the datasets is remarkably high. Spanning all methods in Table 3, the dataset in [81] got its size increased by a factor of around 5, while in [26] the same factor goes up to 7. Adegiba et al. [47] successfully got the number of patches up to 29 times the original size. Each image in [10] remarkably turns into 546 new samples, and the post-augmentation performances improved by almost 25%. Fourteen is the dataset size growing factor in [89], moving from 215 to 3006 ROIs (Regions of Interest). Muduli et al. [35] were able to extend MIAS, DDSM and INbreast, respectively, by 10, 19.2 and 5.4 times their original sizes.

As far as it concerns the data augmentation impact on the methods’ performances, the third column in Table 3 provides details on the accuracy, AUC, sensitivity, and auROC. Unfortunately, due to the lack of experimental results description in the original articles, only some methods can be discussed here. Starting from the fourth row in Table 3, the image co-registration augmentation technique allowed Domingues et al. [81] to gain 33% on accuracy rates. Fundamental transformations such as flipping, rotation, shifting and zooming proved effective in Shi et al.’s method [28], with a gain of 32.3% on the validation accuracy rate over MIAS. Razali et al. [84] obtained post-augmentation performances in breast cancer classification 26% higher. Only rotation, flipping and shearing were applied to INBreast and CBIS-DDSM. The GAN-based data augmentation adopted by Dhiva et al. [10] turned out to be reliable over three datasets: MIAS, DDSM, and INBreast. On the other side, the geometric transformations tested in [30] only slightly hit the target with a small improvement of 1.6% in mammogram classification with VGGNet, GoogleNet and ResNet. Lu et al. [88] reported improvements in Inception V3 sensitivity rates in mass detection over the INBreast dataset that was augmented with geometric transformations plus contrast and brightness adjustments. Rises in sensitivity rates may correspond to drops in false negatives in the detection system. The post-augmentation AUC of the classification system proposed in [31] is higher than the pre-augmentation AUC by 0.21. However, the experimental dataset is private, and the size increase factor obtained with geometric transformations is unknown. If the two advanced techniques adopted in [56,66] struggle to get remarkable classification accuracy rate improvements, the GAN-based method in [55] proved its reliability over two main tasks: mammogram image synthesis and suspicious region detection.

## 6. Conclusions

This paper aims to provide insights into the broader area of mammogram image analysis from a data augmentation perspective. Although some deep learning methods’ performances are excellent, further investigations are necessary to draw a line on the impact of data augmentation on the information generalisation capabilities of supervised deep learning paradigms. Some evidence shows a decisive increase in accuracy rates from basic to advanced augmentation techniques, especially the GANs-based ones. The first sections introduce the main theoretical concepts about the most widely adopted data augmentation techniques in a broader sense. In this work, the techniques mentioned above are surveyed to check out their pros and cons in a specific topic, that is, mammogram image analysis.

The advent of deep learning approaches and the increasingly sophisticated architectures to extract hidden properties from data play a critical role in various computer vision tasks. The main goal here is to discuss specifically the impact of data augmentation techniques on deep learning methods in tasks such as suspicious region detection and classification.

In Table 2 and Table 3, methods from the scientific literature are listed and compared across factors such as task performed, model, dataset, model performance, data augmentation approach. Thanks to the undisputed knowledge inference capabilities of deep learning architectures, most methods reviewed in this paper reach high accuracy rates on their tasks and over some specific datasets. Overall, most DL methods score high in architectural distortion detection, mass detection, density classification, and more generic suspicious region detection. Moreover, the contribution of data augmentation techniques is remarkable, especially to the dataset size increase and accuracy rates improvement. For instance, the tumour classification method proposed in [10] benefits from GAN-based data augmentation, an increased factor of 546 for the dataset. Furthermore, as described in Table 3, the method’s accuracy rate goes up to 94% with an improvement of almost 25% compared to the pre-augmentation performance. Apart from that, our discussion needs to consider that the latter works out mass classification over three different datasets: MIAS, INBreast and DDSM. Moreover, the so-called conventional data augmentation techniques (geometric transformations) allow up to 88% of accuracy, while the more advanced GAN-based techniques outperform them by 6%.

Current and future trends in computer vision see new methods building on self-supervised and semi-self supervised paradigms competing with supervised learning approaches. Purely supervised learning approaches combined with advanced data augmentation should, then, run against self-supervised and semi self-supervised learning methods to balance computational costs, accuracy rates, and information generalisation capabilities.

## Figures and Tables

**Table 1:** Summary of basic and advanced image augmentation techniques.

Sr No.	DA Technique	Sub Category	Label Preserving	Strength	Limitation
1	Geometric Transformation [1,5]	Flipping	No	Good solutions for positional bias present in training data. Easy implementation	Additional memory, Transformation compute cost, Additional training time, Manual observation
		Cropping	Not always		
		Rotation	Not always		
		Translation	Yes		
2	Noise Injection [77]	-	Yes	Allows model to learn more robust	Difficult to decide amount of noise to be added
3	Kernel Filters [1]	-	Yes	Good to generate sharpen and blurred images	Similar to CNN mechanism
4	Mixing Images [78]	-	No	-	Makes not much sense from human perspective. Not suitable for medical images
5	Random Erasing [41]	-	Not always	Analogous to dropout regularization. Designed to combat image recognition challenges due to occlusion, A promising technique to guarantee a network pays attention to the entire image, not a subset of it	Some manual intervention may be necessary depending on the dataset and application
6	Adversarial Training [79]	-	Yes	Help to illustrate weak decision boundaries better than standard classification metrics	Less explored
7	Generative Adversarial Network [80]	-	Yes	GANs generate data that looks similar to original data	Harder to train, Generating results from text or speech is very complex.
8	Neural Style Transfer [60]	-	-	Improves the generalization ability of simulated datasets	Efforts needed to select style, Additional memory, transformation cost

**Table 2:** Summary of articles using Image augmentation.

Ref.	Task Performed	Model	Dataset	Model Performance	Data Augmentation Approach
[24]	AD detection	Deep CNN (Augmented CNN-SW+)	Private	AUC: 0.83 ± 0.14	Rotation by 90, 180 and 270 degrees, mirroring and adding Poisson noise
[8]	AD detection	Deep CNN	MIAS, DDSM, INBreast	Accuracy: 93.75%	Rotation, flipping, shear, scaling, etc.
[25]	Mass detection	Faster R-CNN	CBIS-DDSM	Sensitivity: 0.833 ± 0.038	Horizontal and Vertical Flipping
[63]	Mass detection	mr2NST	mammograms from GE and UIH	-	Neural Style Transfer
[81]	BI-RADS Classification	AlexNet	INBreast	Accuracy: 83.4	Image co-registration
[26]	Tumor detection	Modified AlexNet	MIAS	95.70%	Scaling, horizontal flip, rotation (90, 180, 270)
[27]	Mass Classification	InceptionV3 and ResNet50	DDSM	Accuracy: InceptionV3: 79.6 ResNet50-85.71	Geometric Transformation
[82]	Mammogram classification	Pre-trained CNN Architectures	Private	-	Reflection and Rotation
[28]	BI-RADS classification	CNN	MIAS	Accuracy: 83.6%	Flip, rotation, shift and zoom
[47]	Mammogram Classification	Pre-trained CNN Architectures	MIAS	Accuracy: 99.01%	Gaussian blurring, horizontal flipping, internal refection and mild addition of white noise
[48]	Mass detection	Google Inception-V3	INBreast	ROC: 0.86	Gaussian noise, Flipping, Changing image brightness
[83]	Mass Classification	VGG based DCNN	INBreast, CBIS, BCRP	-	elastic deformations
[10]	Mass Classification	DCNN	MIAS, INBreast, DDSM	Conventional DA techniques: 88% GAN: 94%	GAN
[84]	Mass Classification	AlexNet, InceptionV3	INBreast, CBIS-DDSM	Accuracy: INBreast: Alexnet: 0.9892, InceptionV3: 0.9919 CBIS-DDSM: Alexnet: 0.6138, InceptionV3: 0.8142	rotation, flipping, shearing
[85]	Lesion Classification	ResNet50, VGG16, VGG19	CBIS-DDSM	Accuracy: 90.4%	Geometric transformation, Contrast and brightness adjustment
[86]	Abnormality Classification	Meta Learning, REsnet101	CBIS-DDSM	Accuracy: Meta Learning: 76%, Resnet101: 71%	Geometric transformations
[30]	Mammogram Classification	VGGNet, GoogleNet, Resnet	CBIS-DDSM, MIAS	AUC: 0.932	Geometric transformations
[87]	Mammogram Classification	Residual Networks	INBreast	Specificity: 0.89	Rotation, Translation
[88]	Mass detection	InceptionV3	INBreast	ROC: 0.91	Geometric transformations, Contrast and brightness adjustment,
[31]	Mammogram Classification	Alexnet, Resnet	Private	-	Geometric transformations
[89]	AD detection	Alexnet, SVM	CBIS-DDSM, DDSM, MIAS	Accuracy: 92	Geometric transformations, TTA
[34]	Mammogram detection and classification	YOLO	INBreast	Accuracy: 89.6	Rotation, Flipping
[90]	Build datasets of breast mammography	Alexnet, Densenet, Shufflenet	INBreast	-	Rotation, Flipping
[91]	Mass Detection	Faster R-CNN	OMI-DB	TPR: 0.99 ± 0.03 at 1.17 FPI—malignant 0.85 ± 0.08 at 1.0 FPI—benign	Horizontal Flipping
[92]	Breast cancer diagnosis	Pre-trained CNN Architectures	CBIS- DDSM, BCDR, INBreast, MIAS	F1 Score for MIAS 0.907 ± 0.150	-
[93]	Breast cancer classification	DCNN	MIAS	Accuracy: 90.50	Feature wise data augmentation
[56]	Mass Classification	CNN	DDSM	-	cycle GAN
[33]	Masses Discrimination	GoogleNet	DDSM	Accuracy: 90.38%	Flipping, Cropped-ROI, Gaussian noise
[66]	Image Classification	VGG-16/19	Mini MIAS	-	Crossover technique
[55]	Mass Image Synthesis	GAN	DDSM, Private	-	Contextual Information Based on GANs
[67]	Mass Detection	One-Stage Object Detection Architecture (BMassDNet)	INBreast DDSM	Recall: INBreast: 0.93 DDSM:0.943	Elastic Deformation
[51]	Mass Detection	Fully Convolutional Network	CBIS-DDSM Inbreast	0.8040 PAUC 0.8787 TPR@0.5FPI	Adversarial Learning
[35]	Breast Cancer Classification	Deep CNN	MIAS, DDSM, Inbreast	Accuracy: MIAS: 96.55%, DDSM: 90.68%, INbreast: 91.28%,	Geometric Transformations, Gaussian noise
[36]	Mass Detection	Contrastive Learning, CycleGAN	Inbreast, Private	-	Geometric Transformations
[68]	Mass Classification	Deep CNN	Private	0.760 ± 0.015 for 80% labeled data	Virtual Adversarial Training
[71]	Mass Detection	Eight Object Detection Models	OPTIMAM, Inbreast, BCDR	Out of eight models, DETR [94] could perform well	Cutout and RandConv
[75]	BI-RADS Classification	EfficientNet-B2	Private	Macro F1 score: 0.595	Transparency Strategy
[95]	Mass Detection	Pre-trained CNNs, DenseNet, ResNet, ResNeXt	BCDR	Accuracy: 84%	Geometric Transformations
[96]	Lesion Detection	YOLOv4 Nested Contours Algorithm	INBreast	Sensitivity: 93% by NCA	Geometric Transformations
[97]	Mammogram Density Classification	DenseNet201, ResNet50	MIAS	Accuracy: DenseNet201: 90.47%	Geometric Transformations
[65]	Mass Segmentation	U-Net	DDSM	Sensitivity: 92.32%	Geometric Transformations
[98]	Breast Cancer Detection	Pre-trained CNNs VGG-16, VGG-19, ResNet-50	MIAS	Accuracy: ResNet-50: 71%	Geometric Transformations

**Table 3:** Articles with pre and post augmentation dataset size and model performance.

Ref.	Pre-Augmentation Dataset Size	Post-Augmentation Dataset Size	Post-Augmentation Model Performance
[24]	280 (Mammograms)	345,000 ROIs	-
[8]	5136 ROIs (MIAS), 410 whole images (Inbreast), 322 whole images (MIAS), 55,890 ROIs (DDSM, CBIS)	49,724 ROIs (MIAS), 7914 whole images (MIAS), 1688 whole images (Inbreast), 179,447 ROIs (DDSM, CBIS)	-
[25]	-	8 new labels per image	-
[81]	374	1560 samples	Accuracy improved by more than 33%
[26]	322	2576	-
[82]	3290	26,320	-
[28]	-	-	Rise in validation accuracy from 51.3% to 83.6%
[47]	322	9000	-
[48]	-	-	Increased AUC from 0.78 to 0.86
[83]	-	-	Improved FPI 3.509 (CBIS), 1.864 (BCRP)
[84]	-	-	Rise in accuracy from 0.6026 to 0.8670
[10]	1798	Single image to be augmented into 546 images	Rise in accuracy from 69.85% to 94%
[85]	5257	104,795	-
[30]	-	-	Rise in accuracy from 78.92% to 80.56%
[88]	-	-	Improvement in sensitivity from 0.786 to 0.913
[31]	-	-	Improvement in auROC from 0.62 to 0.73
[89]	215 ROI	3006 ROI	-
[34]	107	428	-
[90]	106	7632	-
[93]	221 (Patches)	1768 Patches	-
[56]	-	-	Improvement in accuracy by 1.4 %
[33]	-	Dataset is expanded by 24 times	-
[66]	-	-	Improvement in accuracy by 1.47%,
[55]	-	-	Improvement in detection rate by 5.03%
[35]	322 (MIAS), 1500 (DDSM), 410 (Inbreast)	3200 (MIAS), 28,800 (DDSM), 2240 (Inbreast)	-
[75]	25,373 (Training Samples)	28,000 (Training Samples)	-
[96]	106	1080	-
[65]	7989	48,659 ROI	-

## Data Availability

Not applicable.

## Abbreviations

The following abbreviations are used in this manuscript:

PRISMA	Preferred Reporting Items for Systematic Reviews and Meta-Analyses
DL	Deep Learning
CNN	Convolutional Neural Network
TL	Transfer Learning
CAD	Computer-Aided Diagnosis
BI-RADS	Breast Imaging Reporting and Database System
ROI	Region of Interest
NST	Neural Style Transfer
GAN	Generative Adversarial Network
AUC	Area Under Curve
ROC	Receiver Operating Characteristic
AD	Architectural Distortion
TTA	Test Time Augmentation
VAT	Virtual Adversarial Training
UIH	United Imaging Healthcare
